# A Rare Case of Acral Lentiginous Melanoma

**DOI:** 10.7759/cureus.38926

**Published:** 2023-05-12

**Authors:** Soham Meghe, Yash Kashikar, Shubham Chopra, Bhushan Madke, Sugat Jawade

**Affiliations:** 1 Dermatology, Jawaharlal Nehru Medical College, Datta Meghe Institute of Higher Education and Research, Wardha, IND; 2 Dermatology, Venereology and Leprosy, Jawaharlal Nehru Medical College, Datta Meghe Institute of Higher Education and Research, Wardha, IND

**Keywords:** histopathology (hp), palm and sole rash, immunohistochemistry staining, dermascopy, melanoma

## Abstract

Acral lentiginous melanoma (ALM) is named so for its site and histological orientation. It is an infrequent form of melanoma that usually presents with lesions on the palms, soles, or nails. Although rare, it's the most commonly discovered subtype of melanoma in the non-Caucasian population, including Africans, Chinese, Koreans, and Latin Americans. It's most likely to be diagnosed in the sixth or seventh decade of life. Acral lentiginous melanoma can clinically mimic ulceration, verrucous lesions, onychomycosis, subungual hematomas, vascular lesions, and infections. Here, we are presenting the case of a 65-year-old male who was admitted to the surgery ward in Acharya Vinobha Bhave Rural Hospital with a chief complaint of a lesion over the plantar surface of his left foot for the last one or two years and was referred to the Department of Dermatology for the same. The lesion was sighted by the patient a long time before his visit to Acharya Vinobha Bhave Rural Hospital. A physical examination showed a blackish, poorly delineated soft tissue lesion on the left heel. An excisional biopsy and proper management were carried out for the patient. Patient education and greater awareness about this tumor and its early detection can serve as important weapons to increase the patient survival rate and prognosis of acral lentiginous melanoma.

## Introduction

Acral lentiginous melanoma (ALM) is an isotype of cutaneous malignant melanoma, primarily found on the soles, palms, or subungual regions [1-3].

Conventionally, it manifests as an asymmetric brown to black macular with irregular edges and could be misdiagnosed as warts or squamous cell carcinoma, sometimes with pyogenic granulomas, chronic fungal paronychia [4], Kaposi's sarcoma, or glomeruloma [5].

Low public awareness of ALM, delayed presentation to health care professionals, and difficulty distinguishing it from normal or traumatic lesions, in addition to the difficulty of obtaining a biopsy from these areas (extremities), contribute to delayed diagnosis and poor disease outcomes [6-9]. Therefore, understanding the characteristics of the early stages of AM is necessary to manage this disease in its early and curable stages.

## Case presentation

A 65-year-old male was referred to the Dermatology OPD of Acharya Vinobha Bhave Rural Hospital by the surgery department, where the patient was initially admitted with the chief complaint of a blackish lesion on the plantar surface of his left foot for one or two years, which had slowly progressed to its current size. It was associated with pain that was insidious in onset and progressive in nature. The patient had no history of hypertension or diabetes mellitus. The patient had no personal or family history of melanoma. The patient denied pruritis, bleeding, or any other systemic symptoms like fever, cough, weight loss, or headache.

**Figure 1:**
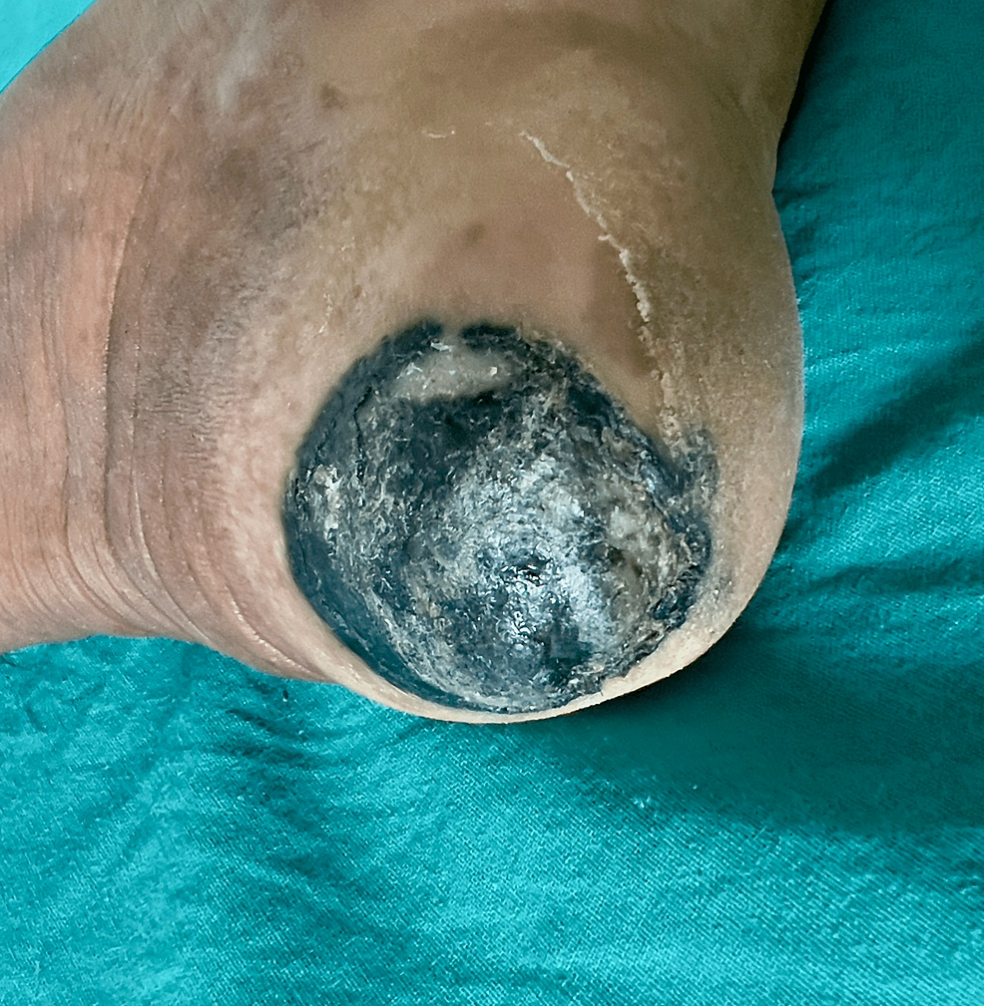
Poorly delineated soft tissue lesion of 3x3.5 cm noted on the left heel region

On general examination, the patient showed vitals within normal limits; there was no sign of pallor, cyanosis, icterus, edema, or lymphadenopathy. The systemic examination was also within normal limits. On physical examination, there was a poorly delineated soft tissue lesion of 3x3.5 cm noted on the left heel region (Figure 1).

Color Doppler of the left lower limb was done (arterial and venous system), and color duplex sonography was performed for the lower limb with a high-frequency probe, which revealed a poorly delineated soft tissue lesion in the left heel region. No abnormality was detected in the Doppler study, and the vasculature appeared normal.

Management

An excisional wedge biopsy was done from the left heel, and the sample was sent for histopathological study and S-100 immunohistochemistry (IHC).

Dermoscopy

**Figure 2:**
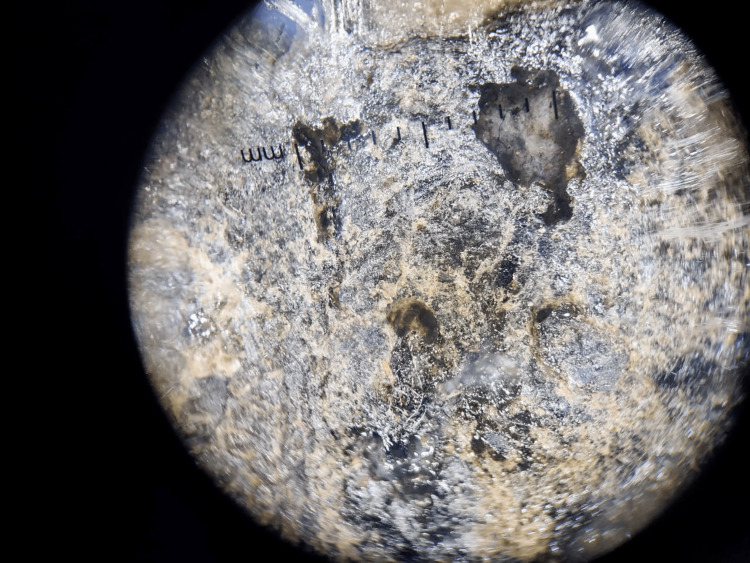
The 12-mm lesion showed a multicomponent pattern with asymmetry, irregular dots and globules, and an irregular fibrillar pattern

The 12-mm lesion showed a multicomponent pattern with asymmetry, irregular dots and globules, and an irregular fibrillar pattern (Figure 2).

Histopathological findings

Irregular, whitish, brownish, or blackish tissue pieces with skin attached measuring 1x1x0.5 cm were detected. The section from the left heel showed extensive proliferation of malignant melanocytes in a pagetoid array suggestive of acral lentiginous melanoma.

**Figure 3:**
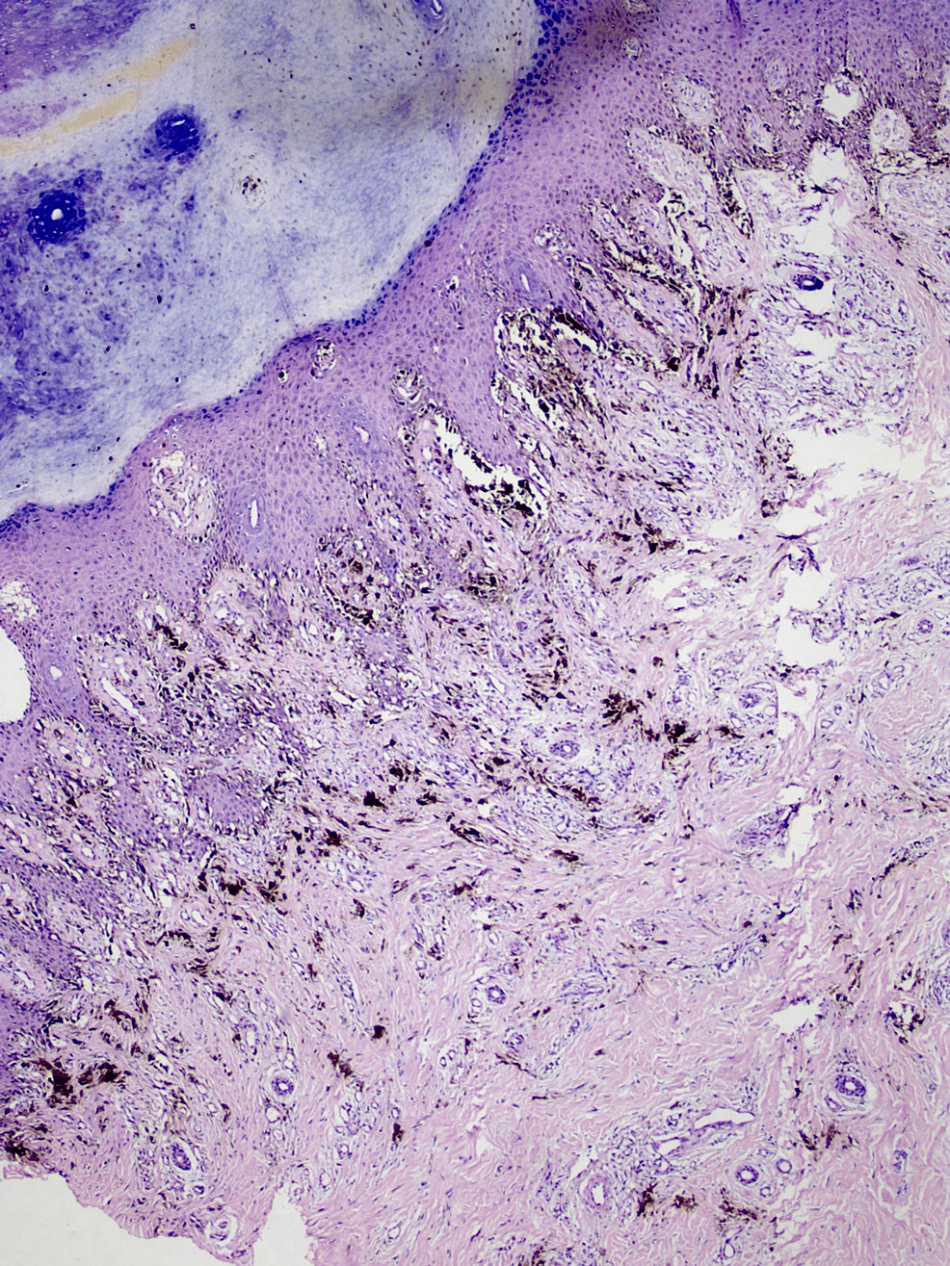
Extensive proliferation of malignant melanocytes in a pagetoid pattern

Breslow thickness was observed to be 2 mm, Clark level was III, growth phase was radical along adnexal structures, lesions were moderately pigmented with no signs of regression, tumor-infiltrating lymphocytes, microscopic satellites, and vascular invasion (Figure 3).

Immunohistochemistry findings

**Figure 4:**
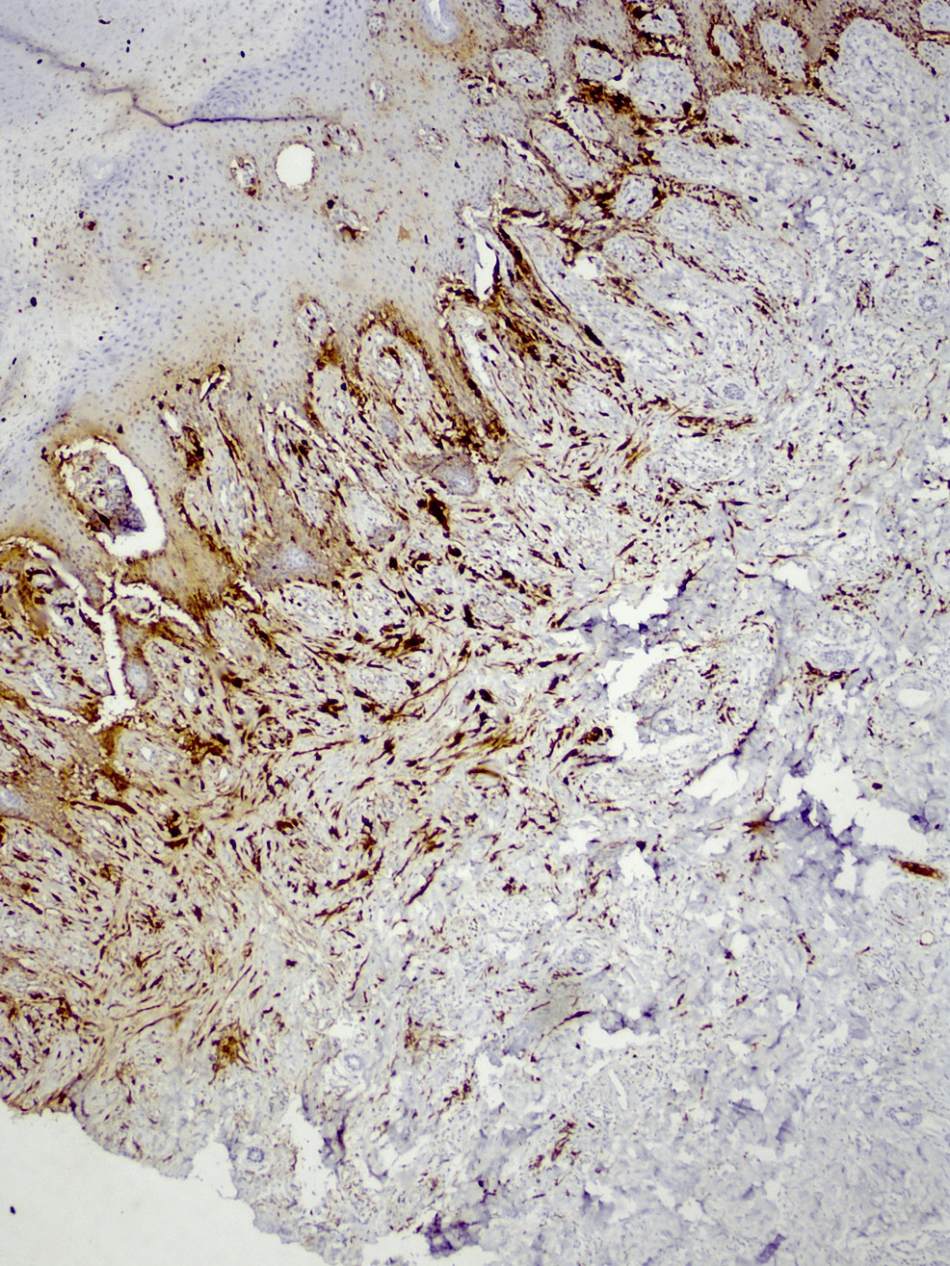
S-100- positive on immunohistochemistry

The sample was S-100-positive on immunohistochemistry (Figure 4).

Postoperative status

The post-operative stay was uneventful, and the patient was discharged and advised to follow up for a positron emission tomography (PET) scan after the IHC report.

## Discussion

Acral lentiginous melanoma was first reported in 1976 by Richard J. Reed and is included as the fourth type of malignant melanoma. Additionally, in 1977, Arrington et al. further outlined the radial component of lentiginous melanoma as well as the distinguishing traits of volar and subungual melanocytic melanoma [10-11]. It has received its name based on its propensity to be stationed at the acral or distal areas of the body. It can also be further classified based on radial or lentiginous growth phases [2].

For the screening of ALM, the clinical criteria known as the ABCDE rule (lesions with asymmetry, border irregularity, color variation, diameter greater than 6 mm, and evolving size, shape, or color) are used as with other types of malignant melanoma. When located in the acral region, the pigmentation follows the skin markings of the palms and soles, resulting in an asymmetrical appearance and an irregular border, even in cases of melanocytic nevus. A parallel ridge pattern (PRP), which can be observed as pigmentation parallel with the ridges of the skin, is the most important finding of ALM in dermoscopy, whereas the histologic feature of ALM is the broad lentiginous growth of melanoma cells [2].

Management of localized ALM follows the same principles as cutaneous melanoma. Biopsy and histological examination will initially confirm the diagnosis and determine the stage of the disease, the extent of surgical resection, and the management of the sentinel lymph node (SLN). In our case, a sentinel lymph node biopsy was not performed as the Breslow’s thickness was only 2 mm [12].

Surgical resection by wide local excision can be the sole treatment needed for early-stage disease, which was followed in the management of the lesion in the present case. However, for higher-stage localized disease(> Stage IIB), adjuvant treatment for one year with immunotherapeutic agents like pembrolizumab or nivolumab is recommended. If a BRAF/MEK mutation is present, then adjuvant treatment with BRAF/MEK inhibitors such as dabrafenib or trametinib could be another option. Acral lentiginous melanoma has a higher percentage of positivity for KIT mutations, and drugs targeting KIT mutations like imatinib have been studied in metastatic settings. Their role in adjuvant treatment has not been established yet [12, 13].

## Conclusions

Acral lentiginous melanoma is a very rare subtype of malignant melanoma but one of the most common forms found in dark-skinned patients. The pathogenesis is not very clear, but it has been shown to involve certain genetic alterations, which include mutations in the BRAF, NRAS, and KIT genes. Acrolentiginous melanoma has a relatively poor prognosis, mainly due to its advanced stage of presentation, which is most likely attributable to delay and misdiagnosis. Greater awareness, a thorough physical examination, proper patient education, and early screening for melanoma in the African and Asian populations are needed to improve the survival outcomes of the disease.
